# Investigation of the Association Between e-Cigarette Smoking and Oral Mucosal Health Status Among Young People: Protocol for a Case-Control Trial

**DOI:** 10.2196/53644

**Published:** 2024-01-26

**Authors:** Siyuan Cheng

**Affiliations:** 1 Massachusetts College of Pharmacy and Health Sciences Boston, MA United States

**Keywords:** oral mucosal lesions, e-cigarette, youth, oral, moth, lesion, lesions, cigarette, cigarettes, smoker, smoking, smokers, smoke, mucosa, mucosal, dental, dentist, dentistry

## Abstract

**Background:**

Given the paucity of current safety studies related to e-cigarettes, there are no definitive studies on whether e-cigarettes cause oral mucosal lesions or even oral cancer. Although it is still undetermined whether e-cigarettes are harmless, an increasing number of teenagers choose to smoke e-cigarettes and believe that they are not harmful to the human body.

**Objective:**

This aims to determine whether e-cigarettes cause damage to the oral mucosa. This study also aims to evaluate the association between e-cigarette smoking and oral mucous membrane lesions in young adults. The objectives are to (1) compare the oral mucosal conditions in participants with and without e-cigarette smoking habits, (2) assess the effect of the amount of e-cigarette smoking on oral mucosal conditions, and (3) assess the effect of the duration of e-cigarette smoking on oral mucosal conditions.

**Methods:**

In this prospective study, 304 youths aged 15 to 24 years (n=152, 50% who smoke only e-cigarettes and n=152, 50% who do not smoke e-cigarettes or cigarettes) will be divided into 2 groups for a controlled study. Whether e-cigarettes cause oral mucosal lesions will be verified by comparing the odds of oral mucosal lesions in the 2 experimental groups. For this experiment, the predefined power is 80% (*P*=.04), and the predefined proportions of groups 1 and 2 are 11% and 2.5%, respectively.

**Results:**

This experiment is at the conceptualization phase and has not yet been carried out. Experimenters have not been recruited and no data have been collected.

**Conclusions:**

e-Cigarettes are still an unfamiliar topic to the public, and it is still unknown whether they can cause damage to the oral mucosa. This experiment aims to find out whether there is a link between the 2. There are still many limitations in this study, such as the lack of categorization of e-cigarettes and the lack of testing methods for oral mucosal status. These limitations are expected to be addressed in the future as the experiment is formally conducted and further optimized.

**International Registered Report Identifier (IRRID):**

PRR1-10.2196/53644

## Introduction

### Background

Oral cavity cancer is the most prevalent head and neck malignancy worldwide [1,2]. This malignant phenotype is often associated with habitual and lifestyle factors, such as tobacco smoking, excessive alcohol consumption, betel nut chewing, and low intake of fruits and vegetables [1]. Due to restrictive government policies and proven negative health effects in many parts of the world, the use of tobacco has declined in recent decades [3]. Electronic cigarettes (e-cigarettes) were invented by a Chinese pharmacist, Hon Lik, in 2003, who envisioned that they would replace conventional cigarettes due to their deleterious effects [4]. e-Cigarette companies claim that handheld devices can provide smokers with the same experience as conventional cigarettes while reducing their negative effects. e-Cigarettes were introduced with the hope that the smoking population would gradually stop using conventional cigarettes and switch to e-cigarettes. With the waning consumption of regular cigarettes, the use of e-cigarettes has surged worldwide, indicating that smokers now consider e-cigarettes viable replacements. Moreover, recent data have indicated that e-cigarette smoking practices are more common among teenagers and young adults [5,6]. However, most people do not take this soaring statistic seriously and allow young people to use e-cigarettes freely. Most people believe that the health hazards of smoking only manifest with increasing age and, thus, concern is unnecessary regarding the use of e-cigarettes by young people.

It is no surprise that young adults are the primary habitual e-cigarette users. The portability of the devices, different flavors with less nicotine, and convenient use of e-cigarettes make them appealing to young people. Data from the 2011-2018 National Youth Tobacco Survey in the United States demonstrated that e-cigarette use among high school students increased from 1.5% in 2011 to 20.8% in 2018 [3]. In 2022, the Centers for Disease Control and Prevention indicated that 2.55 million US middle and high school students reported current (past 30 days) e-cigarette use; nearly 85% of these young people used flavored e-cigarettes, and more than half used disposable e-cigarettes [7]. This increase has also become noticeable in community settings. Unfortunately, adults do not pay enough attention to the use of e-cigarettes, especially in schools.

Current research on e-cigarettes has solely focused on their ingredients and compared satisfaction levels of e-cigarettes with those of regular cigarettes [8,9]. The safety of e-cigarettes, especially regarding the etiology of oral and maxillofacial diseases or other possible intraoral side effects, is still unclear. Moreover, few studies have reported on the adverse effects of e-cigarette smoking [10]. Recent experiments have only indicated that the use of e-cigarettes could impact the balance in the oral microbiome while allowing for the rapid growth of foreign microorganisms [4]. Nevertheless, many studies recommend that individuals replace cigarettes with e-cigarettes irrespective of concerns about their safety.

Smoking causes damage to the oral mucosa as well as lesions that can lead to the progressive development of oral cancer [11]. According to research, smoking can cause “oral mucosal leukoplakia” and a variety of other oral mucosal diseases. Additionally, it has been shown that leukoplakia is the oral mucosal lesion that is most likely to lead to oral cancer [12]. Most of the white spots on the lips occur on the lower lip, in the junction of the middle, and on the outer third of the lip, which is where people usually hold cigarettes; this can explain the relationship between holding cigarettes and white spots on the lip. White spots are precancerous lesions, and approximately 4% to 7% can develop into oral cancer. According to statistics, 93.1% of patients with oral white spots are smokers [13]. The development of oral mucosal lesions is based on the principle that tobacco irritates the oral mucosa (through cigarette smoke and the chemicals in cigarettes), causing it to react adversely to prolonged irritation. e-Cigarettes operate in a similar way to tobacco, which also irritates the oral mucosa through atomization and chemicals.

The only difference between e-cigarettes and cigarettes is simply that e-cigarettes do not contain tar; both contain many chemicals as well as nicotine. e-Cigarettes tend to have more types of chemicals than cigarettes because of the wide variety of flavors.

Given that it is still unknown whether e-cigarettes influence the development of oral mucosal lesions, the possibility that e-cigarettes can cause damage to the oral mucosa and increase the risk of oral cancer similar to tobacco needs to be studied.

To date, no definite investigation of the association between e-cigarettes and oral cavity cancer has been conducted. When available, this study will be the first to determine whether e-cigarettes are mechanistically linked to the development of oral cancer to further educate young users and students on the potential for malignancy due to e-cigarette use. It is assumed that e-cigarettes cause damage to the oral mucosa and oral cancer, which is similar to cigarettes. The following experimental studies and designs will be conducted to test this hypothesis. Therefore, this study aims to investigate the association between e-cigarette smoking and oral mucosal health status among young adults and determine whether e-cigarettes cause damage to the oral mucosa and increase the risk of oral cancer. Compared to that of other similar studies, the methodology of this study is simpler, and the results will be clearly comparable. If the results show that e-cigarettes can cause damage to the oral mucosa similar to that caused by cigarettes, this study could provide strong support for subsequent experiments to determine whether e-cigarettes cause oral cancer.

### Hypothesis

This study hypothesizes that e-cigarettes, in the speculated absence of tobacco, may still induce carcinogenesis. Alternatively, it is suggested that this habit may trigger an imbalance in the oral microbiome that predisposes the mucosal epithelium to oral cavity cancer with other etiological factors. This presupposed risk is likely proportional to the frequency and duration of e-cigarette smoking. The oral mucosal health of participants will be determined by determining the presence of white spots in the mouth (the main feature of the oral mucosa that is damaged by smoking behavior).

### Primary Outcome

The primary outcome is the effect of e-cigarette smoking on oral mucosal conditions.

### Research Significance

The results of this study will provide preliminary evidence of the malignant potential of e-cigarettes in the deterioration of oral mucosal conditions or exclude them as a cause of oral carcinogenesis. If a direct association is observed, the results of this study would be vital to inform e-cigarette users of the harmful and even carcinogenic nature of e-cigarette smoking, despite e-cigarettes being considered tobacco free. In addition, this would help guide the implementation of legislative policies to bring awareness to the additional risks associated with the use of e-cigarettes and vaporizers. Moreover, these findings, if positive, will pave the way for future research on chemical products that may be present in e-cigarettes that are directly involved in the malignant transformation of oral keratinocytes.

## Methods

### Trial Registration

Given that this study is at this stage of conceptualization and has received no support or sponsorship from any organization at this time, this experiment has not yet been applied for trial registration. The registration will be done in the future when support is received from the relevant organizations.

### Ethical Considerations

This study has not yet been submitted to the ethical review board for assessment, mainly due to lack of financial support and lack of assistance from large organizations. We emphasize that this is an independent study and is still at the research protocol stage. As the project has not received sufficient financial support, we are unable to cover the costs of applying for evaluation by the ethics review committee at this time. At this stage, we are working to ensure that the study design meets ethical standards and will seek possible review and approval at a future stage. We understand and value participant rights and will take appropriate measures to ensure the ethical and legal nature of the study.

### Study Design

The adoption of a case-control, prospective, observational study design is motivated by the relatively low incidence of oral mucosal lesions within the general population. This design allows for a focused exploration of the relationship between e-cigarette smoking and oral mucosal health.

Case-control design is particularly suitable for investigating rare outcomes such as oral mucosal lesions, as it efficiently compares individuals with the outcome of interest (cases) to those without (controls).

A prospective design involves following participants over time, allowing for the collection of data on exposures and outcomes as they occur. By prospectively tracking participants, the study can gather real-time information on e-cigarette smoking habits and observe the development of oral mucosal lesions, providing a temporal sequence crucial for establishing causation.

An observational design is chosen over an experimental one due to ethical considerations and the nature of the research question. Since randomly assigning participants to smoke e-cigarettes for an extended period is ethically challenging, an observational approach allows for the examination of naturally occurring exposure to e-cigarette smoking in real-world settings.

The low incidence of oral mucosal lesions in the general population necessitates a design that efficiently targets and investigates this specific outcome. By focusing on a population at risk (e-cigarette smokers) and carefully selecting controls, the study maximizes its ability to detect and understand the potential impact of e-cigarette smoking on oral mucosal health.

### Study Population

According to the United Nation definition, people between the ages of 15 and 24 years are defined as young people [14]. This age group coincides with the rapidly growing population of e-cigarette smokers, which is not a concern to mainstream society. Therefore, the 15- to 24-year-old group has been selected as the study population. American participants between 15 and 24 years of age will be recruited irrespective of their sex, race, occupation type, and socioeconomic status and divided into “e-cigarette case” and “control” groups. The selection criteria for each group are as follows.

The exclusion criteria for the two groups (the following criteria will be applicable in both groups and do not need to be listed repeatedly) are (1) participants with histologically diagnosed recurrent oral cavity cancer whose primary tumors occurred outside the recruitment timeframe (2 years before the first diagnosis); (2) participants with synchronous solid or hematological malignancies in other regions at the time of oral cavity cancer diagnosis; (3) participants with a genetic predisposition to oral cavity cancer, including those with Fanconi anemia, systemic lupus erythematosus, and dyskeratosis congenita; (4) participants who meet the inclusion criteria but are unwilling to participate in the study after detailed information has been provided; and (5) participants with severely debilitating systemic conditions that preclude participation in the study.

### Selection of the “e-Cigarette Case” Group

#### Inclusion Criteria

A prospective study among experimental participants who smoked e-cigarettes over the last 210 days or 7 months will be conducted. According to the article “Effects of Duration of Electronic Cigarette Use,” the average duration of use among e-cigarette smokers is 210 days or 6.8 months [15]. The same criteria will be used in this study to standardize the duration of use for both the conventional cigarette smoking population and the e-cigarette smoking population.

#### Exclusion Criterion

The exclusion criterion is participants who smoke tobacco.

### Selection of Controls

Inclusion criteria are individuals without a history of smoking e-cigarettes, drinking alcohol, and smoking tobacco at the time of recruitment.

Exclusion criteria are participants who smoke e-cigarettes and tobacco.

### Recruitment Strategy

#### Overview

The recruitment strategy, targeting customers at e-cigarette stores and pedestrians, is strategically aligned with the study’s focus on e-cigarette users. The inclusion of community selection for matching controls, if quotas are not met, demonstrates flexibility in the recruitment approach. The disclosure of convenience and nonprobability sampling methods ensures transparency, thereby acknowledging the limitations inherent in these methods. A nuanced understanding of participant selection challenges is crucial for the accurate interpretation of study findings.

#### Community Selection Details

If quotas for controls are not met through the initial recruitment strategy, a community selection approach will be considered. This involves identifying and recruiting controls from community settings, ensuring a diverse representation.

### Clinical Recruitment Procedures

#### e-Cigarette Store Recruitment

The e-cigarette store recruitment consists of 3 stages, which are as follows:

1. Approach: e-cigarette stores will be approached to seek their cooperation in the recruitment process.
2. Informed consent: store owners and managers will be provided with detailed information about the study objectives, procedures, and ethical considerations. Upon agreement, consent forms may be obtained from the store owners to allow recruitment on their premises.
3. Participant identification: e-cigarette users within the specified age range will be approached, and the study will be explained to them. Interested individuals will be given detailed information about the study and their consent will be sought.

#### Pedestrian Recruitment

The pedestrian recruitment consists of 2 stages, which are as follows:

1. Approach: pedestrians in high-traffic areas will be approached with an invitation to participate in the study.
2. Informed consent: similar to the e-cigarette store recruitment, detailed information will be provided to potential participants, and consent will be obtained before proceeding with any study-related activities.

#### Community Selection (if Necessary)

If quotas for controls are not met through the initial recruitment strategy, community selection may be considered.

1. Approach: community settings such as local community centers or public spaces will be identified. Consent from relevant authorities and community leaders will be sought.
2. Participant identification: controls meeting the study criteria will be approached in these settings, and recruitment will follow the same informed consent procedures.

### Sampling Methods

Convenience and nonprobability sampling methods will be used for the recruitment of cases and controls. The detailed sample size determination is shown in Multimedia Appendix 1 [16]. Briefly, the predefined power is 80% (*P*=.04), and the predefined proportions of the case and controlled groups are 11% and 2.5%, respectively. In all, 304 youths aged 15 to 24 years (n=152, 50% who smoke only e-cigarettes and n=152, 50% who do not smoke e-cigarettes or cigarettes) will be recruited.

### Data Collection: Questionnaire

Interviewer-administered questionnaires will be used to collect data on demographic, lifestyle, and socioeconomic variables for the cases and controls. The questionnaire tool will comprise 2 parts: exposure information (part A) and sociodemographic information (part B). A life grid sheet will be used during interviews to efficiently collect retrospective information on lifestyle habits and other risk factors. To reduce observer bias, interviewers will not initially be informed about the aim of the research. Once cases are identified and interviewed, matching controls will be recruited and interviewed. Answers provided by participants will be converted to objective scores after data collection by the investigator (SC). The details on the parts of the questionnaire are as follows.

Exposure information (part A) will include (1) oral mucosal health status (whether white spots are present), (2) smoking habits, (3) type of products used, (4) e-cigarette smoking status (yes or no and current or previous), (5) age of onset, (6) duration of smoking, (7) pattern of smoking duration (continuous or intermittent), (8) frequency of smoking (daily, weekly, and occasionally), (9) number of cartridges (frequency), and (10) Tobacco consumption. Sociodemographic information (part B) will include (1) age, (2) sex, (3) occupation type, (4) education level, (5) income, and (6) ethnicity.

### Questionnaire Validity and Reliability

Before the deployment of the questionnaire for field use, face validity and qualitative content validity will be determined by a panel of experts in oral and maxillofacial surgery, dental public health, biostatistics, and laypersons. The internal consistency of the scales used to measure e-cigarette smoking exposure will be determined using Cronbach α, with a minimum α value of .70 indicating good and acceptable reliability.

### Data Analysis

Descriptive statistics will be used for all binary, categorical, and continuous variables and expressed as tables, texts, and figures. Bivariate analysis will then be performed for relevant variables. The Shapiro-Wilks test will be performed for continuous variables to determine whether they follow the Gaussian distribution. Afterward, the independent 1-tailed *t* test and 1-way ANOVA will be used; otherwise, the Mann-Whitney *U* test and Kruskal-Wallis test will be performed. For cross-tabulation of 2 categorical variables, the chi-square test will be used. Variables that do not fulfill the assumption of this test will be analyzed using Fisher exact test. Comparisons with *P*=20 will be used to implement multivariable analysis using multiple logistic regression. Odds ratios and 95% CIs will be determined for e-cigarette smoking and other individual factors. For all analyses, *P*<.05 will be used to denote statistical significance. Statistical analyses will be performed using the SPSS (version 27; IBM Corp).

The control and e-cigarette groups will be subjected to a *t* test (assuming the data meet the normality criteria and do not meet the criteria for the rank sum test) to verify whether e-cigarettes cause harm.

## Results

This experiment is at the conceptualization phase and has not yet been carried out. Experimenters have not been recruited and no data have been collected.

## Discussion

In examining the potential negative impact of e-cigarettes on the oral mucosa, this study deliberately focused its investigation on this specific aspect, excluding other potential confounding factors that might influence the results. Notably, variables such as alcohol consumption, daily routines, and gender differences were not considered in the experimental design. While these factors were omitted from this study, they were duly documented in the questionnaire phase, laying the groundwork for future investigations to delve into these variables. This strategic choice aimed to isolate the primary relationship between e-cigarette use and oral mucosal health, with the intention to refine and expand the scope in subsequent research endeavors.

The findings of this study align with existing literature on e-cigarettes, which suggests a correlation between the chemicals present in e-cigarettes and cytotoxicity, leading to damage to the oral mucosa. This corroborates with prior research, providing further evidence of the potential harm associated with e-cigarette use [10,16,17]. The recognition of this association holds significant implications for public health, emphasizing the need for awareness campaigns and regulatory measures to mitigate the adverse effects on oral health. Moreover, the study acknowledges the diverse landscape of e-cigarettes in the United States, encompassing various brands and flavors, each containing distinct chemical compositions. Given this complexity, it is acknowledged that the study does not comprehensively analyze all individual chemicals present in different e-cigarettes. This limitation prompts a call for future experiments to undertake a more nuanced examination of the diverse chemical profiles within e-cigarettes to better understand their distinct impacts on oral mucosal tissues.

Despite the valuable insights provided by this study, certain limitations should be acknowledged. The decision to exclude factors such as alcohol consumption and daily routines may have implications for the generalizability of the findings. In addition, the complexity of e-cigarette compositions poses a challenge, as the study did not extensively investigate the myriad chemicals present in different e-cigarette products. Future research endeavors should consider a more comprehensive approach, encompassing a broader range of variables and a detailed analysis of the chemical constituents of various e-cigarettes.

In conclusion, this study contributes valuable evidence to the growing body of knowledge on the potential negative impact of e-cigarettes on oral mucosal health. By focusing on a specific aspect while recognizing its limitations, this research paves the way for future investigations to build upon these findings. The implications extend beyond the immediate scope of oral health, emphasizing the broader need for public health interventions and regulatory measures in response to the evolving landscape of e-cigarette use.

## Appendix

Multimedia Appendix 1 Sample size determination.

## Data Availability

Data sharing is not applicable to this paper as no data sets were generated or analyzed during this study.
