# Supplementary material for: Investigation of the Association Between e-Cigarette Smoking and Oral Mucosal Health Status Among Young People: Protocol for a Case-Control Trial
Source: JMIR Res Protoc. 2024 Jan 26;13:e53644. doi: 10.2196/53644 (PMC10858415; doi:10.2196/53644)
Supplement: Multimedia Appendix 1 [file resprot_v13i1e53644_app1.docx]

### Sample Size Determination

Since there are too few available data from experiments on e-cigarettes and the oral mucosa, most of which cannot be matched to the purpose of this study, the design of the data model and the number of experimental participants in this trial will be determined by referencing previous case‒control studies on tobacco use and the oral mucosa. In the article “Smoking and oral leukoplakia: A cross-sectional study among the Gond Tribe in Madhya Pradesh” [16], 11% of smokers and 2.5% of nonsmokers developed leukoplakia. This ratio will be used as the research parameter for this study. A parallel two-group design will be used to test whether the proportion (P1) of Group 1 (treatment group) differs from that (P2) of Group 2 (control group) (H0: P1 - P2 = 0 versus H1: P1 - P2 ≠ 0). The comparison will be made using a two-sided, two-sample t test, with a type I error rate (α) of 0.05. The control group proportion (P2) is assumed to be 0.025. To detect a proportional difference (P1 - P2) of 0.085 (P1 of 0.11) with 80% power, the number of subjects needed in both Group 1 (treatment group) and Group 2 (control group) is 136. The sample size was computed using PASS 2023, version 23.0.2.

According to the sample size calculation software "PASS", if the power is 80%, P=.04, and the proportions of Groups 1 and 2 are 11% and 2.5%, respectively, then the final total number of experimental participants will be 272 (136 participants each in the control and experimental groups). Considering a 10% attrition rate, the final total number of experimental participants will be 304 (152 participants per group).
